# Association of IGF‐1 gene rs2195239 polymorphism with the risk and clinical features of gastric cancer in a Chinese Han population

**DOI:** 10.1002/jcla.23436

**Published:** 2020-07-05

**Authors:** Amirhosein Meisami, Amin Jalilvand

**Affiliations:** ^1^ Emergency Medicine Kermanshah University of Medical Sciences Kermanshah Iran; ^2^ Department of Biology Faculty of Science University of Mohaghegh Ardabili Ardabil Iran

**Keywords:** gastric cancer, IGF‐1 gene, letter to editor, Rs2195239 polymorphism, susceptibility

Dear Editor,

We read with the great interest the published paper by Wang et al^1^. They performed a research to examine the association of rs2195239 polymorphism with the risk and clinical features of gastric cancer in a Chinese Han.

In abstract, it was mentioned that “Stratified analyses suggested that the significant association was shown in the females.” Besides, in Table 1, the authors stated that the number of males and females in Control group were 225 and 193, respectively. Nevertheless, in Table 3, the total of male and female in control groups were 298 and 257, respectively. These contradictory data can challenge the statistical analysis and, as the result of this error, challenge the validity of the significant association between gastric cancer with rs2195239 polymorphism in the female population.

Despite the error as mentioned above, this research had valuable results in assessing the role of IGF‐1 gene rs2195239 polymorphism on the gastric cancer incidence.

## CONFLICTS OF INTEREST

The authors declare that they have no conflict of interest.
